# GGQ methylation enhances both speed and accuracy of stop codon recognition by bacterial class-I release factors

**DOI:** 10.1016/j.jbc.2021.100681

**Published:** 2021-04-20

**Authors:** Shreya Pundir, Xueliang Ge, Suparna Sanyal

**Affiliations:** Department of Cell and Molecular Biology, Biomedical Center, Uppsala University, Uppsala, Sweden

**Keywords:** ribosome, translation, release factor, translation termination, methylation, GGQ motif, accuracy, a.u., arbitrary units, ATP, adenosine triphosphate, BOP, BODIPYTM 576/589, CD, circular dichroism, cryo-EM, cryo-electron microscopy, EF, elongation factor, FRET, fluorescence resonance energy transfer, IF, initiation factor, k_cat_, maximal rate of catalysis, k_cat_/K_M_, catalytic efficiency, K_M_, Michaelis–Menten constant, M-M, Michaelis–Menten, MD, molecular dynamics, mRF, methylated release factor, RC, release complex, RF, release factor, rNTP, ribonucleoside triphosphate, rRNA, ribosomal ribonucleic acid, RT-PCR, real-time polymerase chain reaction, Tm, melting temperature

## Abstract

Accurate translation termination in bacteria requires correct recognition of the stop codons by the class-I release factors (RFs) RF1 and RF2, which release the nascent peptide from the peptidyl tRNA after undergoing a “compact to open” conformational transition. These RFs possess a conserved Gly-Gly-Gln (GGQ) peptide release motif, of which the Q residue is posttranslationally methylated. GGQ-methylated RFs have been shown to be faster in peptide release than the unmethylated ones, but it was unknown whether this modification had additional roles. Using a fluorescence-based real-time *in vitro* translation termination assay in a stopped-flow instrument, we demonstrate that methylated RF1 and RF2 are two- to four-fold more accurate in the cognate stop codon recognition than their unmethylated variants. Using pH titration, we show that the lack of GGQ methylation facilitates the “compact to open” transition, which results in compromised accuracy of the unmethylated RFs. Furthermore, thermal melting studies using circular dichroism and SYPRO-orange fluorescence demonstrate that GGQ methylation increases overall stability of the RF proteins. This increased stability, we suspect, is the basis for the more controlled conformational change of the methylated RFs upon codon recognition, which enhances both their speed and accuracy. This GGQ methylation-based modulation of the accuracy of RFs can be a tool for regulating translational termination *in vivo*.

Termination is an important step of translation during which the nascent peptides are released from the ribosome. It should be efficient for allowing fast turnover of the translation machinery, and at the same time, accurate to avoid accumulation of the potentially inactive and toxic, truncated, or overlong proteins. Protein synthesis terminates when a translating ribosome encounters one of the three stop codons (UAA, UAG, and UGA) on the mRNA at the ribosomal A site. These codons are recognized in a semispecific manner by the class-I release factors (referred hereafter as RFs) in bacteria, namely release factor 1 (RF1) and release factor 2 (RF2). UAA codon is read by both RF1 and RF2. But, UAG is read specifically by RF1 and UGA by RF2 (1). In eukaryotes, all three stop codons are read by a common class-I RF named eRF1 (2). The class-I RFs in bacteria depend on the class-II RF RF3 for their fast dissociation from the ribosome after peptide release (3).

RF1 and RF2 possess a common universally conserved Glycine–Glycine–Glutamine (GGQ) motif (4) in the tip of domain III, which is involved in release of the nascent peptide by ester bond hydrolysis from the peptidyl tRNA at the peptidyl transferase center (PTC) of the ribosome (5, 6, 7, 8, 9, 10, 11). Mutations of the GGQ motif render RF1 and RF2 inactive in peptide release (8, 12, 13). In both prokaryotes and eukaryotes, the amide group of the Gln (Q) residue in the GGQ motif is posttranslationally methylated at the N^5^ position by a methyltransferase enzyme encoded by *prmC* (also known as HemK) (*for review see* (14, 15, 16, 17)). Knocking out *prmC* gene is not lethal but results in slow phenotypic growth (18). Earlier *in vitro* (10, 14, 19, 20) and *in vivo* studies (18, 21) demonstrated that GGQ methylation significantly enhances the catalytic activity of the RFs. The enhancement is more pronounced for some particular amino acids (20). Using X-ray crystallography and cryo-electron microscopy (cryo-EM), it has been recently shown that the GGQ methylation helps this motif in acquiring a stable conformation in the PTC, which in turn facilitates efficient peptide release by the RFs (10).

Accuracy of translation termination depends on correct stop codon recognition by the class-I RFs. RF1 and RF2 possess different sequence motifs for recognition of the specific stop codons at the decoding center (DC) of the ribosome. Earlier studies based on mutational analysis demonstrated that RF1 uses a Proline-X-Threonine/Alanine/Valine (PXT) motif and RF2 uses a Serine–Proline–Phenylalanine (SPF) motif located in the domain-II for specific recognition of their cognate stop codons (22, 23). Later, molecular dynamics (MD) simulations-based studies (24) identified additional residues beyond the PXT and SPF motifs as crucial for correct stop codon recognition by RF1 and RF2. Among these the role of Arg213 of RF2 has been studied extensively by mutagenesis and fast kinetics (25).

Structural studies of the RFs with X-ray crystallography and cryo-EM have revealed that RF1 and RF2 exist in two different conformations in free state and when bound to the ribosome (5, 6, 7). In the crystal structures, corresponding to the free state, RF1 and RF2 are seen in a compact form, with PXT/SPF motif and GGQ motif separated by a distance of 25 Å (Fig. 1*A*) (26, 27). In contrast, the class-I RFs mimic aminoacyl-tRNAs on the ribosome and adopt an open conformation spanning ∼75 Å from the DC on the small subunit to the PTC on the large subunit (Fig. 1*B*) (5, 7, 11, 28, 29, 30, 31). It was proposed earlier that RF1 and RF2 confer the catalytically active open conformation upon correct codon recognition after binding to the ribosome (Fig. 1*C*) (5). The visual evidence for this hypothesis, however, appeared only very recently through time-resolved cryo-EM, which demonstrated a “compact to open” transition of RF1 and RF2 on the ribosome after proper recognition of the stop codon (32). Contemporary cryo-EM studies have also reported different intermediate conformations (between fully compact and open) of RF1 and RF2 on the ribosome (33, 34, 35). Moreover, kinetics of the “compact to open” transition has been followed by state-of-the-art fluorescence resonance energy transfer (FRET) (36) and *in silico* simulations (9). Altogether these studies decipher a near complete mechanistic picture of translation termination by the bacterial class-I RFs, where the stop codon recognition and conformational change relate to the accuracy in termination, whereas the peptide release governs the speed of the process (Fig. 1*C*).

**Figure 1:**
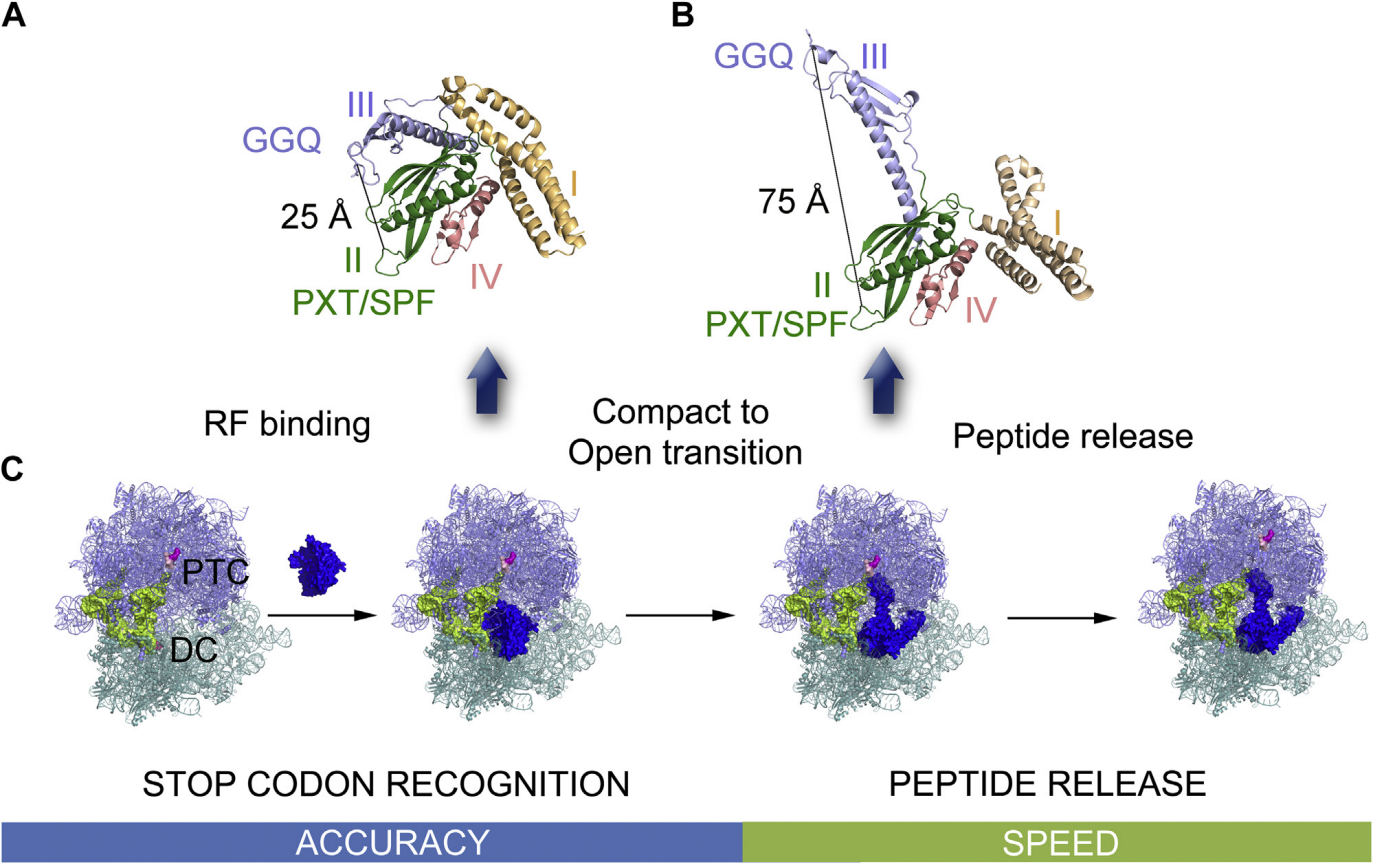
**Class-I release factor-mediated steps of translation termination, which govern the speed and accuracy of the process.***A* and *B*, represent compact and open conformations of RFs, respectively, with the domains I, II, III, and IV colored in *yellow*, *green*, *purple*, and *pink*. In the closed state, the distance between the GGQ and the PXT/SPF motifs is 25 Å, which increases to 75 Å in the open state on the ribosome. *C*, the scheme describes the main events of translation termination where the RFs bind to the ribosome with an empty A-site harboring a stop codon in the compact state. The RFs undergo “compact to open” transition upon recognition of the stop codon and accommodation in the A-site. Lastly, the peptide is released from the peptidyl tRNA by participation of the GGQ motif of the RFs in the open state. The first two steps contribute to accuracy of the process, whereas the catalytic peptide release state governs speed of translation termination.

The first quantitative analysis of accuracy of stop codon recognition by RF1 and RF2 was performed by Ehrenberg and colleagues. With precise biochemical experiments, they demonstrated the degree of preference of RF1 and RF2 for their cognate stop codons over the near-cognate codons for efficient peptide release (37). Their conclusion was that accuracy in termination depends entirely on the selective recognition of the stop codons by the RFs. However, the new finding that RF1 and RF2 open up on the ribosome upon correct stop codon recognition and thereby become catalytically active (Fig. 1) (32), adds an additional regulatory step in the accuracy process. In addition, it raises the question whether GGQ methylation has any role in modulating this conformational transition and thus, affecting the accuracy of stop codon recognition by RF1 and RF2. Recently, Zeng *et al*. (10) reported kinetic parameters for peptide release by the methylated and unmethylated RFs on the stop codons, but the accuracy impact has not been explored.

Here, using a fluorescence-based *in vitro* peptide release assay in stopped-flow, we show that GGQ methylation in RF1 and RF2 enhances both catalytic speed and accuracy of stop codon recognition. Furthermore, by pH titration, we demonstrate that the lack of GGQ methylation expedites the “compact to open” transition of the RFs, thereby making the unmethylated RFs less accurate in peptide release. Finally, by biophysical characterization of the unmethylated and methylated RFs, we find that the GGQ methylation aids in overall stability of the proteins. This increased stability, we suspect, is the basis for more controlled conformational change in the methylated RF1 and RF2 upon codon recognition, which enhances their speed and accuracy.

## Results

### Kinetics of peptide release by mRF2 and RF2 on cognate (UAA, UGA) and near-cognate (UAG, UGG) codons

**Figure 2:**
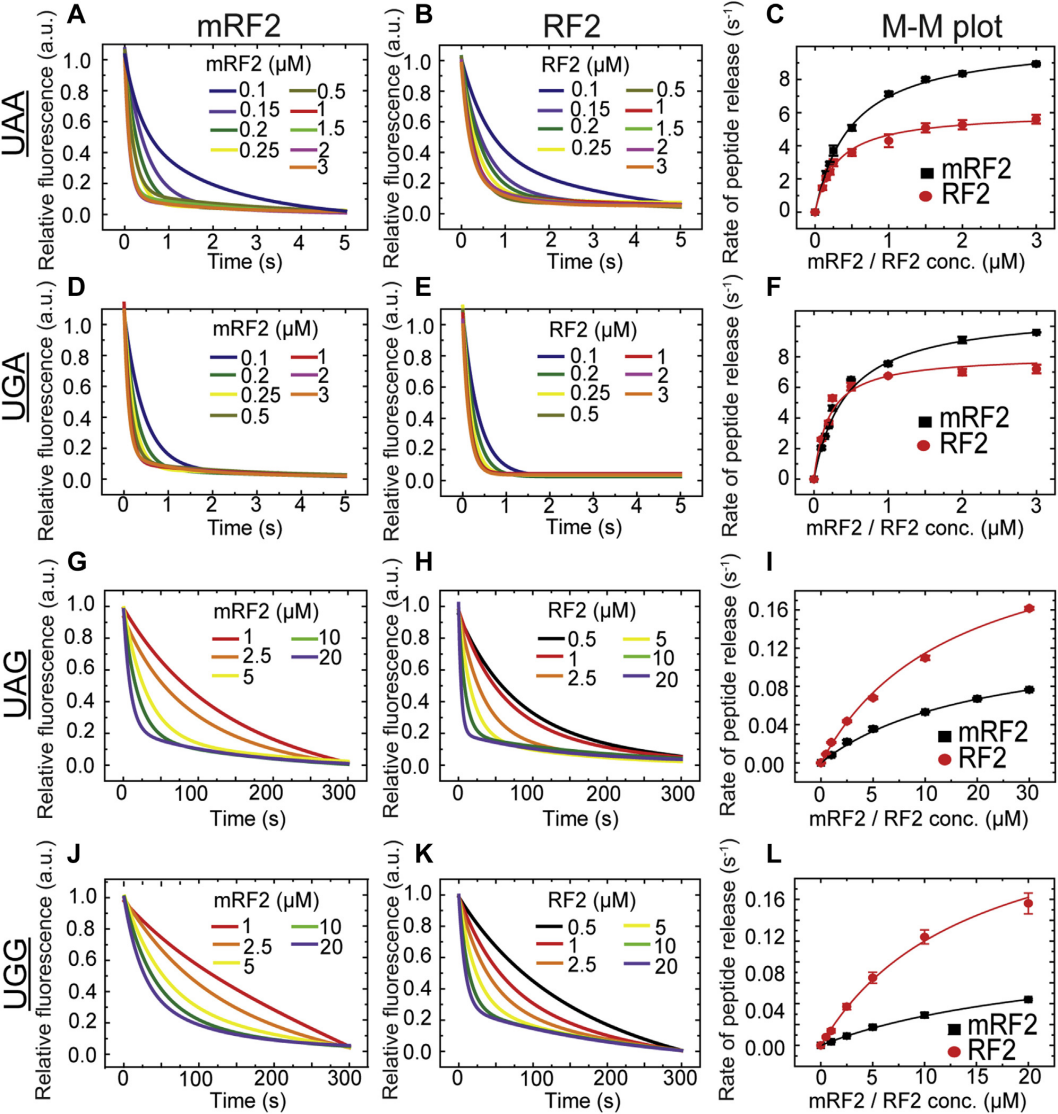
**Stopped-flow-based fluorescent BOP-Met-Leu-Leu tripeptide release kinetics by mRF2 and RF2 on RCs containing cognate/near-cognate codons.** The *left* (*A*, *D*, *G*, and *J*) and the *middle panels* (*B*, *E*, *H*, and *K*) present the time courses of BOP fluorescence change in arbitrary units (a.u.) due to BOP-Met-Leu-Leu tripeptide release from cognate RC_UAA_ and RC_UGA_ and near-cognate RC_UAG_ and RC_UGG_ (0.1 μM), with increasing concentrations of mRF2 (*left*) and RF2 (*middle*). The *right panels* (*C*, *F*, *I*, and *L*) represent the Michaelis–Menten plots (M-M plot) demonstrating dependence of the observed peptide release rates (derived from the titration curves in the *left* and *middle panels*), on the concentration of mRF2 and RF2 on corresponding codons (labeled in the *left* for each row). The kinetic parameters (*k*_*cat*_, *K*_M_, and *k*_*cat*_/*K*_M_) are derived by fitting the curves with hyperbolic function. The error bars indicate SEM from minimum three sets of independent experiments.

For peptide release assay, we used a ribosomal release complex (RC), which harbored in the P-site, a peptidyl tRNA carrying fluorescent BODIPY 576/589 (BOP) labeled Met-Leu-Leu tripeptide, and in the A-site, one of the three stop codons or the UGG codon specific for tryptophan (Trp), on the mRNA. The RF2 variants, methylated (mRF2) and unmethylated (RF2), were added in increasing concentration to the RC with either cognate UAA and UGA codons or near-cognate UAG and UGG codons, and the time course of peptide release was monitored by following decrease in BOP fluorescence in a stopped-flow instrument (Fig. 2, left and middle panel). The rates of peptide release were determined by fitting the fluorescence curves with exponential functions (see Experimental procedures for details). The observed rates plotted against concentration of the RFs were fitted with hyperbolic function following Michaelis–Menten equation (Fig. 2, right panel), from which the kinetic parameters *k*_*cat*_, *K*_M_, and *k*_*cat*_/*K*_M_ were derived.

Our kinetics data (Table 1), consistent with earlier reports (19, 20, 37, 38), show that mRF2 releases peptide from cognate RC_UAA_ and RC_UGA_ with the maximal catalytic rate (*k*_*cat*_) of about 10 s^−1^, which is about 1.7-fold higher than that of RF2 (Fig. 2, *C* and *F*). However, the increase in *k*_*cat*_ is accompanied by an equivalent increase in *K*_M_ by mRF2, thereby resulting in similar catalytic efficiencies (*k*_*cat*_/*K*_M_) of mRF2 and RF2 on the cognate UAA and UGA codons (Table 1). This result, in complete agreement with the previous report (38), confirms that the methylation of GGQ does not have any impact on catalytic efficiency of peptide release by RF2 on the cognate codons. However, when the same assay was performed with near-cognate RC_UAG_, RF2 showed about twofold higher *k*_*cat*_ than mRF2 with almost no change in *K*_M_ (Fig. 2*I*). Thus, catalytic efficiency of RF2 on UAG codon is about 2.5-fold higher than mRF2 (Table 1). In line with this result, RF2 shows about fourfold higher catalytic efficiency of peptide release than mRF2 on Trp coding UGG codon (Table 1). On RC_UGG_, RF2 releases peptide with 2.5-fold higher *k*_*cat*_ than mRF2 (Fig. 2*L*). Interestingly, mRF2 shows 1.5-fold higher *K*_M_ than RF2 on RC_UGG_. Thus, together these two parameters cause a bigger difference in the catalytic efficiency of RF2 *versus* mRF2 on UGG than on UAG codon (Table 1).

**Table 1:** The Michaelis–Menten parameters for peptide release by the methylated and unmethylated RFs on cognate and near-cognate codons

Codon	A site codon	Release factor	*k*_*cat*_ (s^−1^)	*K*_M_ (μM^−1^)	*k*_*cat/*_*K*_M_ (s^−1^ μM^−1^)
Cognate	UAA	mRF2	10.4 ± 0.04	0.49 ± 0.007	21.2 ± 0.40
		RF2	6.0 ± 0.17	0.30 ± 0.01	20.0 ± 0.008
	UGA	mRF2	10.7 ± 0.03	0.38 ± 0.009	28.2 ± 0.36
		RF2	6.8 ± 0.19	0.28 ± 0.04	24.3 ± 0.007
Near-cognate	UAG	mRF2	0.13 ± 0.01	15.5 ± 1.76	0.008 ± 0.0006
		RF2	0.28 ± 0.004	14.7 ± 0.10	0.019 ± 0.0004
	UGG	mRF2	0.10 ± 0.04	22 ± 1.4	0.0045 ± 0.0001
		RF2	0.25 ± 0.03	14.4 ± 0.11	0.017 ± 0.0006
Cognate	UAA	mRF1	8.3 ± 0.06	0.06 ± 0.005	138.3 ± 0.003
		RF1	2.0 ± 0.04	0.03 ± 0.03	66.7 ± 0.005
Near-cognate	UGA	mRF1	0.16 ± 0.004	2.7 ± 0.03	0.059 ± 0.004
		RF1	0.12 ± 0.003	2.6 ± 0.26	0.046 ± 0.004

**Figure 3:**
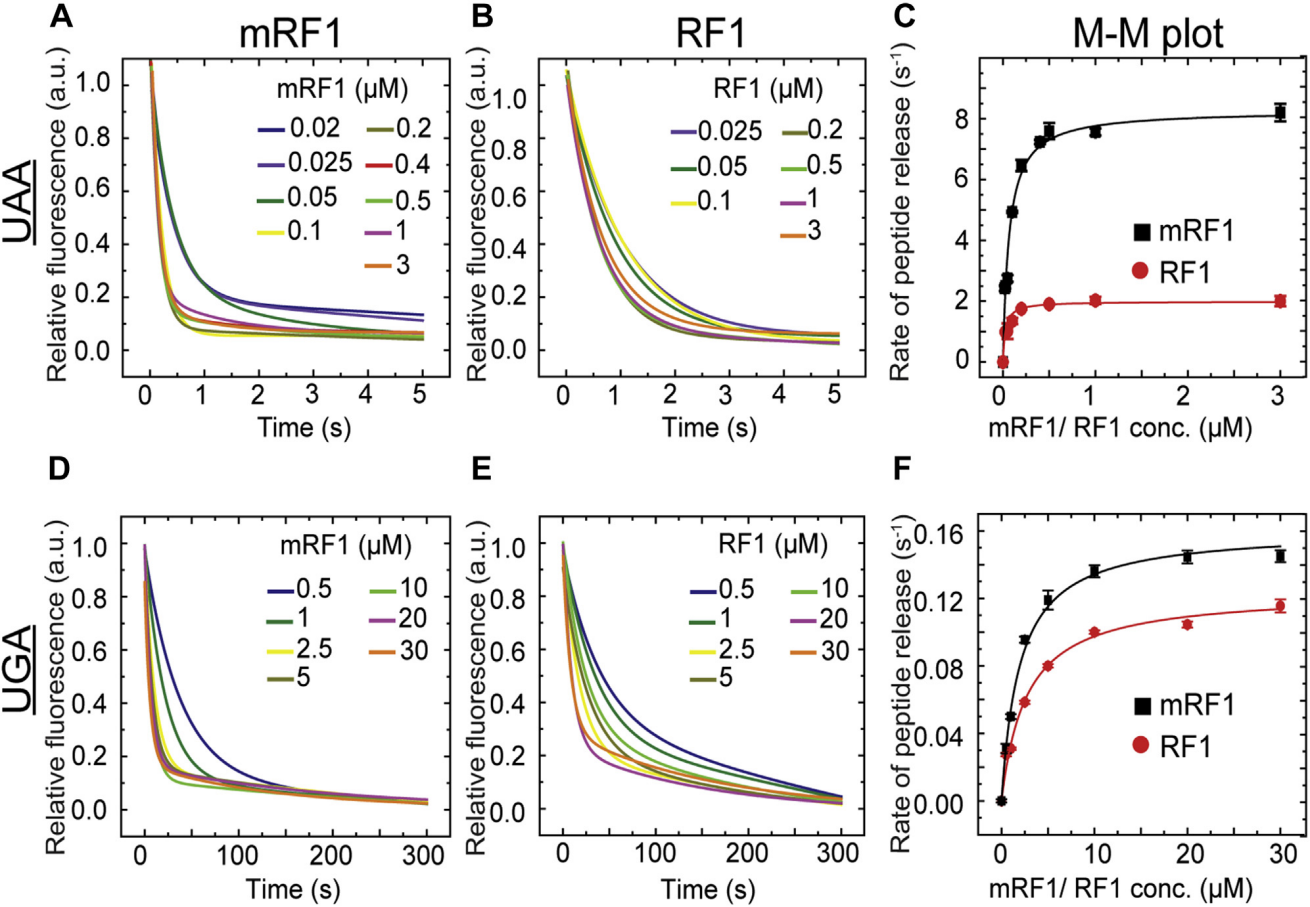
**Stopped-flow-based fluorescent BOP-Met-Leu-Leu tripeptide release kinetics by mRF1 and RF1 on RC containing cognate/near-cognate codons.** The first two panels present the time courses of BOP fluorescence change due to BOP-Met-Leu-Leu tripeptide release from cognate RC_UAA_ and near-cognate RC_UGA_ (0.1 μM), with increasing concentrations of mRF1 (*leftpanels*: *A* and *D*) and RF1 (*middlepanels*: *B* and *E*) in arbitrary units (a.u.). The *rightpanels* (*C* and *F*) are the Michaelis–Menten plots (M-M plot) representing dependence of the observed peptide release rates (derived from the titration curves in the *left* and *middle panels*), on the concentration of mRF1 and RF1 on corresponding codons (labeled in *left*). The kinetic parameters of peptide release (*k*_*cat*_, *K*_M_, and *k*_*cat*_/*K*_M_) by mRF1 and RF1 are derived by fitting the curves with hyperbolic equation. The error bars indicate SEM from minimum three sets of independent experiments.

The values are obtained from M-M plots (Figs. 2 and 3, right panels) with SEM (Standard Error of Mean) estimated from at least three independent sets of experiments.

As expected, mRF2 and RF2 show similar *k*_*cat*_ and *K*_M_ values on the two cognate codons UAA and UGA (Table 1). However, both mRF2 and RF2 show slightly higher catalytic efficiency (*k*_*cat*_/*K*_M_) for UGA (28.2 ± 0.36 and 24.3 ± 0.007) than UAA (21.2 ± 0.4 and 20 ± 0.008). Our results clearly demonstrate that mRF2 has higher catalytic activity, but similar catalytic efficiency of peptide release on cognate codons. However, on near-cognate codons, RF2 shows higher catalytic activity as well as catalytic efficiency than mRF2.

### Kinetics of peptide release by mRF1 and RF1 on cognate (UAA) and near-cognate (UGA) codons

We compared mRF1 and RF1 for their catalytic efficiency in peptide release from the RCs containing cognate (UAA) and near-cognate (UGA) codon using the stopped-flow-based Bop-Met-Leu-Leu tripeptide release assay (Fig. 3, left and middle panel). The observed rates estimated from the time course of the peptide release are plotted against mRF1 and RF1 concentration and fitted with hyperbolic function to obtain the Michaelis–Menten parameters (*k*_*cat*_, *K*_M_, and *k*_*cat*_/*K*_M_) (Fig. 3, right panel).

Our kinetic data show that mRF1 possesses 4.2-fold higher catalytic activity than RF1 for peptide release from the cognate RC_UAA_, with *k*_*cat*_ 8.3 s^−1^ (Fig. 3*C*, Table 1). Along with the increase in *k*_*cat*_, mRF1 also shows a twofold increase in *K*_M_, thereby resulting in twofold higher catalytic efficiency (*k*_*cat*_/*K*_M_) than RF1 for peptide release on the cognate UAA codon (Table 1). This result confirms that mRF1 has higher catalytic efficiency than RF1 for peptide release on the cognate UAA codon. However, on the near-cognate RC_UGA_, mRF1 and RF1 show similar catalytic activity with *k*_*cat*_ 0.16 s^−1^ and 0.12 s^−1^ respectively, with no change in *K*_M_ (Fig. 3*F*). Thus, mRF1 and RF1 show similar catalytic efficiency of peptide release on UGA codon (Table 1).

Thus, our results demonstrate that methylation of RF1 results in significantly higher catalytic activity and catalytic efficiency of peptide release on the cognate codon (Table 1).

### Accuracy of the class-I RFs

To determine the accuracy of the RFs, we introduce a parameter called “A value,” which is the ratio of their catalytic efficiencies of peptide release (*k*_*cat*_/*K*_M_) on cognate *versus* near-cognate codons (37). In other words, the A value describes the relative preference of the RFs for the cognate codon (reference codon) over the near-cognate codon (or essentially any other codon). Here, we have used the major stop codon UAA as the reference codon.

With regard to the two cognate codons UAA and UGA, both mRF2 and RF2 show slightly higher preference for UGA with the A values 0.74 ± 0.017 and 0.83 ± 0.0004, respectively. However, significantly larger A values are obtained when catalytic efficiencies are compared between UAA and near-cognate UAG and UGG codons. The A values summarized in Table 2 show that mRF2 favors peptide release on UAA over UAG by a factor of 2625 ± 203 and over UGG by a factor of 4200 ± 116. These values are in close agreement with the values previously reported by Freistroffer *et al*. (37). The unmethylated RF2, however, shows less discrimination for the near-cognate codons, about 1000 ± 25 for both UAG and UGG. Thus, comparison of the respective A value shows that mRF2 is 2.6 and 4.2 times more discriminative than RF2 for near-cognate UAG and UGG codons, respectively (Table 2). These results thus demonstrate that mRF2 is not only more efficient than RF2 for peptide release, but also more accurate for correct recognition of the cognate stop codons.

**Table 2:** Accuracy of stop codon recognition by methylated and unmethylated release factors

Release factor 2	Cognate *versus* Cognate	←Cognate *versus* near-cognate→			
	A(_UAA/UGA_)	A(_UAA/UAG_)	A(_UAA/UGG_)	Release factor 1	A(_UAA/UGA_)
mRF2	0.74 ± 0.017	2625 ± 203.13	4200 ± 116.00	mRF1	2300 ± 153.33
RF2	0.83 ± 0.0004	1000 ± 20.00	1000 ± 30.00	RF1	1340 ± 107.20
mRF2/RF2	0.9	2.6	4.2	mRF1/RF1	1.72

The data are presented as “A values.” A = (*k*_*cat*_/*K*_M_) _cognate_/(*k*_*cat*_/*K*_M_) _near-cognate_ with SEM estimated from at least three independent sets of experiments.

We have also estimated the accuracy of mRF1 and RF1 for UAA (cognate) *versus* UGA (near-cognate) codons. The A values summarized in Table 2 show that mRF1 favors UAA codon over UGA, 2300 ± 153 times. In comparison, RF1 shows lesser preference for UAA than UGA, 1340 ± 107 times. Thus, mRF1 is 1.7 times more discriminative than RF1 for the near-cognate UGA codon (Table 2).

It is interesting to compare the basis of the A values for the RF1 and RF2 variants. The mRF2 and RF2 show similar *k*_*cat*_/*K*_M_ values on the cognate codons, but mRF2 shows lower *k*_*cat*_/*K*_M_ values than RF2 on the near-cognate codons (Table 1). In the contrary, mRF1 shows higher *k*_*cat*_/*K*_M_ on the cognate codon than RF1, but similar *k*_*cat*_/*K*_M_ on the near-cognate codon (Table 1). Thus, higher accuracy of mRF2 originates from its relatively lower efficiency of near-codon recognition than RF2. In contrast, higher accuracy of mRF1 is due to its relatively higher efficiency of cognate codon recognition than RF1. However, in both cases the methylated RFs show higher accuracy than the unmethylated forms.

### Determination of the rate-limiting step for peptide release by RFs and mRFs using pH titration

**Figure 4:**
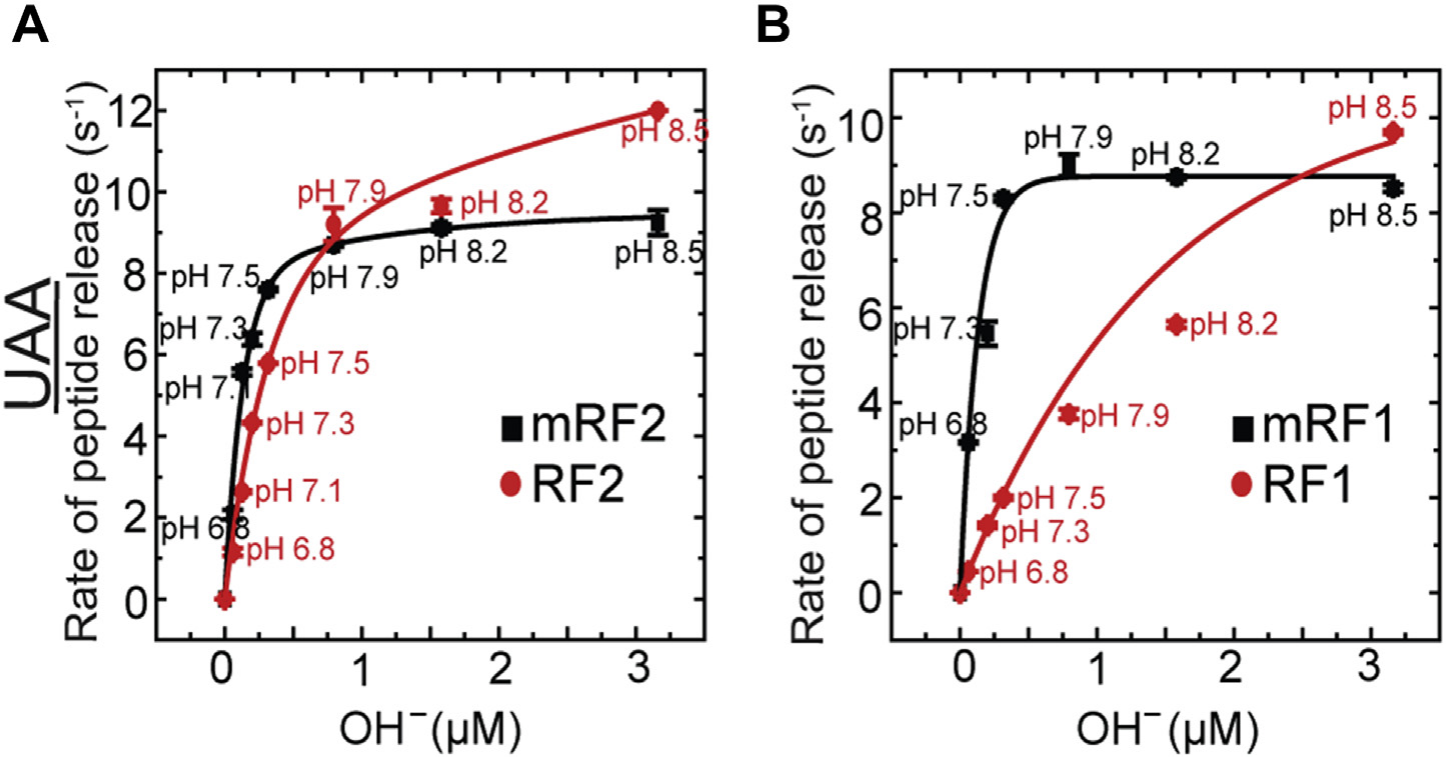
**pH dependence of *k***_***cat***_**of BOP-Met-Leu-Leu tripeptide release by methylated and unmethylated RFs.** The maximal rates of peptide release by mRF2 and RF2 (*A*) and mRF1 and RF1 (*B*) on RCU_AA_ are plotted as a function of OH^−^ ion concentration.

As suggested by current literature (19, 32), the “compact to open” conformational transition is the critical step, which makes the RFs catalytically active for peptide release (Fig. 1*C*). Based on our accuracy measurements, we hypothesized that the RFs lacking GGQ methylation might undergo this transition more easily than mRFs, which compromise their accuracy of peptide release on cognate codons. To test that hypothesis, we followed pH dependence of the maximal rate of peptide release (*k*_*cat*_) by mRFs and RFs on RC_UAA_ (Fig. 4). It has been shown that peptide release by the class-I RFs is rate limited by the chemistry of ester bond hydrolysis at low pH and by a pH-independent conformational change step at high pH (19). Our aim was to find out whether the methylated and unmethylated RFs undergo transition from the “pH-dependent” to the “pH-independent” state in similar pH or not.

The stopped-flow-based BOP-Met-Leu-Leu tripeptide release assay was conducted with RC_UAA_ in HEPES-polymix buffer with pH adjusted to 6.8 to 8.5. To ensure maximal rates, saturating concentration of RFs (4 μM) was used. In good agreement with the published data (19), our results show that *k*_*cat*_ for mRF2 increased linearly from 2.2 s^−1^ at pH 6.8 to 9 s^−1^ at pH 7.9, after which it did not increase further (Fig. 4*A*). Interestingly, *k*_*cat*_ for RF2 did not saturate in this pH range. Instead, it showed a biphasic linear increase, with slightly different slopes before and after pH 7.5. At pH 8.5 it released peptide with a rate 12 s^−1^, which is even higher than mRF2 at that pH (Fig. 4*A*).

For mRF1 and RF1 the difference was more drastic, but the overall trend was same as mRF2 and RF2. For mRF1, the *k*_*cat*_ increased linearly from 3.2 s^−1^ at pH 6.8 to 8.7 s^−1^ at pH 7.9 after which it saturated. In contrast, RF1 showed pH-dependent biphasic linear increase in the entire pH range tested here and produced higher *k*_*cat*_ (10 s^−1^) than mRF1 (8.5 s^−1^) at pH 8.5 (Fig. 4*B*).

Thus, both mRF2 and mRF1 showed “pH-dependent” to “pH-independent” transition of *k*_*cat*_ at ∼pH 7.9, while for RF2 and RF1 the *k*_*cat*_ increased in a pH-dependent manner even at higher pH. Thus, for RF2 and RF1, the pH-dependent catalytic step remained “rate limiting” and thus “slower” than the pH-independent conformational change step for the entire pH range tested here. Conversely, for mRF2 and mRF1, the catalytic step was rate limiting only at pH less than 7.9. These results suggest that in contrast to the methylated RFs, unmethylated RFs presumably undergo “compact to open” transition faster than the catalytic step even in high pH. It means that RF2 and RF1 are inherently more prone to “compact to open” transition than mRF2 and mRF1.

### Comparison of the conformational stability of mRF2 and RF2 by thermal melting

To check whether the lack of methylation affects the overall stability of RFs in free state, thermal melting profiles of mRF2 and RF2 were recorded using (i) Circular Dichroism (CD) and (ii) SYPRO-orange thermal shift assay.

**Figure 5:**
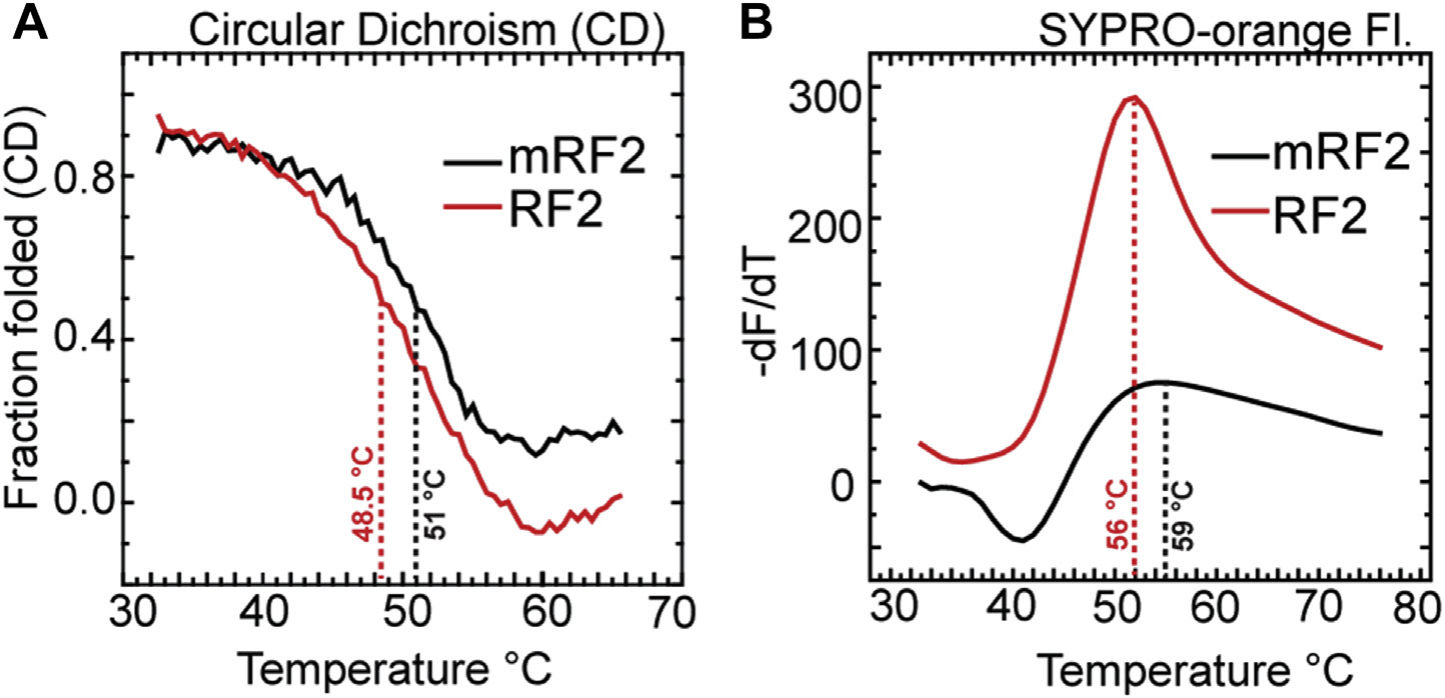
**Thermal melting profiles of mRF2 (*black traces*) and RF2 (*red traces*) for comparison of their structural stability.***A*, thermal melting of secondary structure of mRF2 and RF2 followed by CD at 220 nm. The plots represent the fraction of folded proteins, as estimated from the ratio of relative change in ellipticity for a given temperature change over the total change in ellipticity (see Experimental procedures for details). The transition midpoints are recorded as T_m_. *B*, SYPRO-orange thermal shift assay to follow tertiary structure melting of mRF2 and RF2. The plots represent the first derivative of the fluorescence emission at 569 nm as a function of temperature. The peak of the derivative plots is considered as T_m_.

CD provides information about secondary structure of a protein. When mRF2 and RF2 were subjected to thermal melting in a JASCO J-1500 CD spectrometer, both showed gradual loss of the intrinsic helicity at 220 nm with increase in temperature. The fraction of the folded protein was estimated from the ratio of the CD value at a given temperature with the total change in CD. As shown in Figure 5*A*, the fraction of the folded mRF2 and RF2 decreased with increase in temperature and the main transition for both was observed after 40 °C. The melting temperature (T_m_) estimated from the midpoint of the transition was 51 °C for mRF2 and 48.5 °C for RF2 (Fig. 5*A*). Although the observed difference was small, the results were highly reproducible. Also, big difference in the CD-based melting temperature was not expected from the difference of a single methyl group between the two RF2 variants.

Further, SYPRO-orange thermal shift assay was conducted using an RT-PCR machine to compare the thermal stability of the tertiary structures of mRF2 and RF2. The SYPRO-orange fluorescence changes upon binding to the hydrophobic patches of the protein, thereby reflecting on the changes in the tertiary structure of the proteins. The first derivative of the change in the SYPRO-orange fluorescence was plotted for mRF2 and RF2, as a function of temperature (Fig. 5*B*). The T_m_, derived from the peak value of the derivative curves for mRF2 was 59 °C and for RF2 was 56 °C (Fig. 5*B*). However, RF2 showed noticeably greater fluorescence change than mRF2, suggesting that the dye had larger access to its hydrophobic patches than to mRF2. These results indirectly argue in favor of a relatively flexible structure of RF2 than mRF2.

## Discussion

The role of GGQ methylation in the bacterial RFs has been studied extensively with *in vivo* genetics and *in vitro* biochemistry (10, 14, 18, 19, 20, 21). These studies unanimously reported increased efficiency of peptide release by virtue of GGQ methylation of the RFs. This is understandable as the GGQ motif places itself in PTC, directly in the site of hydrolysis of the peptidyl tRNA. It is nicely demonstrated by a recent cryo-EM structure (10), which showed that the methylated GGQ motif adopts a highly ordered conformation, which facilitates its interaction with A76 of the peptidyl tRNA as well as with U2506, A2451, and A2452 nucleotides of 23S RNA. However, there was no knowledge so far whether GGQ methylation can also influence other steps of translation termination.

Our results clearly demonstrate the effect of GGQ methylation in the class-1 RFs on both catalytic activity and accuracy of translation termination. On the basis of the A value calculation (Table 2), we infer that accuracy originates from the lower efficiency of recognition of the near-cognate codons than the cognate codons by the RFs. When the catalytic efficiencies were compared for peptide release on cognate *versus* near-cognate codons, the unmethylated RFs were two- to four-fold less accurate than the methylated RFs by performing less efficient discrimination between a cognate and a near-cognate codon. This applies not only to the near-cognate stop codons, but also to the Trp codon encoded by UGG. However, question remains that how lack of GGQ methylation may affect accuracy of translation termination, when the methylation site is located far from the stop codon recognition site on the ribosome.

We revisited the conformational cycle of the RFs during translation termination to seek an answer to the question above. The RFs are seen in the compact state (Fig. 1*A*) in complex with the methyltransferase HemK encoded by the gene *prmC*, which methylates the GGQ motif (17). The RFs also bind to the ribosome in the compact state (33, 34, 35). In this state, the GGQ motif on domain III positions itself closer to the domain II, thereby forming the II–III–IV super domain (26). Upon codon recognition, rearrangement of the switch loop between the domains III and IV directs the opening of domain III carrying the GGQ motif to reach the PTC (30). It has been suggested that stop codon recognition in the DC brings 23S rRNA closer to the RFs, thereby stabilizing their open and active state (39). This “compact to open” transition of the RFs acts as a “check-point” for the RFs before peptide release. Since the GGQ motif shows close interaction with domain II in the compact state, we asked whether GGQ methylation can influence the conformational transition of the RFs from the compact to the open conformation.

It has been shown the rate of the catalytic peptide release step by RFs escalates with an increase in pH until it becomes faster than the pH-independent compact to open transition step, which then becomes rate limiting (19). We conducted peptide release assays at different pH to find out at which pH the transition from pH-dependent to pH-independent kinetics happens in RFs in comparison to mRFs. Our results show that for both mRF1 and mRF2, the pH-independent “compact to open” transition step becomes rate limiting at pH higher than 7.9. In contrast, for unmethylated RF1 and RF2, the rate of peptide release keeps increasing in a pH-dependent manner even at pH 8.5. This means that for the unmethylated RFs, the catalytic step remains rate limiting. Thus, the lack of GGQ methylation likely induces error in RF1/2 by promoting easier transition to the open state, which allows them to bypass the “check-point,” irrespective of the cognate or near-cognate codon.

We further asked whether the lack of GGQ methylation perturbs the overall stability of the RFs. For that we performed thermal melting experiments with mRF2 and RF2 and followed the transition with CD and SYPRO-orange fluorescence. The T_m_s estimated from the transition midpoints for mRF2 was slightly higher than RF2 in both assays suggesting higher stability of the mRFs. However, much higher binding of SYPRO-orange to RF2 than the same concentration of mRF2 indicated that RF2 allows higher access to the dye to its hydrophobic patches. Thus, unmethylated RFs likely have lesser structural rigidity than mRFs, which may explain why the RFs undergo easier transition from compact to open conformation than the mRFs.

Recent report suggests that ribosomal dynamics during RF1 and RF2 interaction with RF3 play a major role in translation termination (40). It is known that following peptide release, RF3 mediates dissociation of RF1 and RF2 from the ribosome (41). Although most of the RF3 interactions are confined at domain I of RF1 and RF2 (42), it is an interesting question whether structural dynamics of RF1/RF2 can influence the process. Since our results indicate that GGQ methylation modulates structural flexibility of the RFs, whether or not it also has impact on the RF3-mediated steps subsequent to peptide release remains to be seen in future.

In summary, we suspect that a relatively less-rigid structural architecture of the RFs in the absence of GGQ methylation allows them to undergo easier transition from the compact to the open state, thereby compromising their accuracy for codon-specific peptide release. However, the accuracy-loss effects are mild compared with the loss of catalytic activity associated with the lack of GGQ methylation in RFs. Since *prmC* is not an essential housekeeping gene and its expression varies in different growth conditions, we suspect that GGQ methylation may work like a tool for regulation of speed and accuracy of translation termination, especially in the stress conditions. In a cell when survival becomes a big question, the unmethylated RFs probably manage to terminate translation even on the near-cognate codons with their compromised accuracy, which would rescue the ribosomes from sequestration. We propose that this can be one of the physiological implications of GGQ methylation in the bacterial RFs.

## Experimental procedures

### Buffer and the components of the translation system

70S ribosome and tRNA^fMet^ were purified from bacteria *Escherichia coli* MRE600 using a standard protocol (43). The *E. coli* initiation factors (IF1, IF2, and IF3) and the elongation factors (EF-Tu, EF-Ts, and EF-G) were prepared from the respective C-terminal His-tagged constructs, cloned in pET21a or pET24b, overexpressed in *E. coli* BL21(DE3), and further purified using Nickel-affinity chromatography HisTrap HP(Cytiva, former GE Healthcare) as described earlier (43). The RFs and mRNA preparations are described below. All experiments were conducted at 37 °C in HEPES-polymix buffer (pH 7.5) containing 5 mM HEPES (pH 7.5), 5 mM Mg(OAc)_2_, 1 mM dithioerythritol (DTE), 5 mM NH_4_Cl, 0.5 mM CaCl_2_, 100 mM KCl, 1 mM spermidine, and 8 mM putrescine (44). The reaction mixes also contained energy pump components 1 mM ATP, 10 mM phosphoenol pyruvate (PEP), 0.05 mg/mL pyruvate kinase (PK), 0.02 mg/mL myokinase (MK) unless mentioned otherwise.

### Preparation of methylated and unmethylated RFs

The *prfA* gene encoding RF1 and the *prfB,246T>A* gene encoding RF2Ala246 (referred in the manuscript only as RF2) were cloned into pET24a vectors, with C-terminal (His)_6_ tag. It has been shown that the C-terminal (His)_6_ tag does not affect the activity of the RFs in peptide release (13). These plasmids were transformed into *E. coli* BL21-Gold(DE3) strain for overexpression of the unmethylated RFs. For the methylated RFs, the plasmids were cotransformed with the plasmid pACYCDuet-1 *prmC* expressing HemK methyltransferase (16). The unmethylated and methylated RF1 and RF2 proteins were overexpressed by addition of 1 mM IPTG at OD_600_ = 0.6 at 37 °C for 3 h. The proteins were purified by affinity chromatography by using a HisTrap High-Performance column (Cytiva, former GE Healthcare) and stored in HEPES-polymix buffer (pH 7.5).

To confirm the state of methylation, the purified RF variants (RF1, RF2, mRF1, and mRF2) were treated with Trypsin/Lys-C Mix (Promega), subjected to mass spectrometry using LC-orbitrap MS/MS, and were analyzed using MaxQuant 1.5.1.2 software as well as Proteome Discoverer 1.4 (Thermo Fisher Scientific) at mass spectrometry–based proteomics facility at Uppsala University. The results summarized in Figs. S1 and S2 confirmed 100% methylation of mRF1 G235 and mRF2 G252 (Figs. S1*A* and S2*A*) and also complete absence of the methylation in RF1 and RF2 (Figs. S1*B* and S2*B*).

### Preparation of the mRNAs

The reverse complementary oligo sequences were ordered from MERCK with sequences 5′- AAGCTTGAAATTAATACGACTCACTATAGGGAATTCGGGCCCTTGTTAACAATT*AAGGAGG*TATTAA**ATGCTGCTGTAA**GAATTC-3′ and 5′-GAATTC**TTACAGCAGCAT**TTAATA*CCTCCTT*AATTGTTAACAAGGGCCCGAATTCCCTATAGTGAGTCGTATTAATTTCAAGCTT-3′.

The underlined part constitutes T7 promoter. The bold and underlined part of the mRNA sequence encodes Met-Leu-Leu-stop (UAA/UAG/UGA) or Trp (UGG) codon. The part in italics represents a strong Shine-Dalgarno sequence. The oligonucleotides (1 μg/μL) were mixed and heated at 94 °C for proper annealing. Later, the reaction was cooled to room temperature resulting in a double-stranded template for transcription. The mRNA was transcribed at 37 °C using T7 RNA polymerase and transcription buffer. The transcription buffer contains 200 mM HEPES (pH 7.5), 30 mM MgCl_2_, 30 mM DTE, 2 mM Spermidine, 2.5 mM rNTPs, and 5 μg DNA template. The transcribed mRNA is then extracted by phenol and chloroform (1:1) treatment. The extracted mRNA was precipitated with 75% ethanol for 2 h and then subjected to purification on HiLoad 26/60 Superdex 75 prep grade gel filtration column (45). The concentration of the purified mRNA was determined, and further, the activity of the mRNAs was determined by titrating the mRNAs in dipeptide formation assay as described in (46).

### Preparation of BOP-Met-tRNA^fMet^

The fluorescence dye used in the assay, BODIPY 576/589 (BOP) was purchased from Thermo Scientific. Charging of Met-tRNA^fMet^ was performed in the reaction with 80 μM tRNA^fMet^, 150 μM Met, 2 U/μL Met-tRNA^Met^ synthetase in a buffer containing 30 mM HEPES (pH 7.5), 1X polymix, 1 mM DTE, 2 mM ATP, 20 mM PEP, 2 mM Mg(OAc)_2_, 0.05 mg/mL PK, 0.01 mg/mL MK. After incubation of the charging mix for 30 min at 37 °C, the charged tRNA was extracted by phenol and chloroform (1:1) treatment and later precipitated with ethanol. The precipitated tRNA was dissolved in water.

Labeling of the Met-tRNA^fMet^ was performed in 100 mM NaHCO_3_ (pH 8.0) by mixing Met-tRNA^fmet^ and BOP in excess, for overnight in dark at 4 °C. The labeled tRNA was extracted by phenol (pH 4.3) and chloroform (1:1) treatment, then precipitated with ethanol, and dissolved in ddH_2_0.

The BOP-Met-tRNA^fMet^ was purified using HPLC system (Waters) equipped with LiChospher WP 300 RP-18 column in line UV and fluorescence detectors. A linear gradient of 12% to 54% methanol in a buffer containing 20 mM NH_4_Ac (pH 5.0), 5 mM MgCl_2_, and 400 mM NaCl was used to elute BOP-Met-tRNA^fMet^. Fractions corresponding to the peak of UV (260 nm) and fluorescence (Ex. 565 nm, Em. 610 nm) were pooled together, concentrated in Amicon Ultra-15 centrifugal filters (10 kDa cutoff), and stored as aliquots at −80 °C.

### Preparation of release complex (RC)

The RC, a stalled ribosome carrying a peptidyl tRNA with Met-Leu-Leu (MLL) tripeptide in the P site and a stop codon (UAA/UAG/UGA) in the A site, was prepared with the initiation mix (IM) and elongation mix (EM) in HEPES-polymix buffer (pH 7.5) containing energy pump components. IM contained 2 μM 70S ribosomes, 3 μM mRNA, 2 μM BOP-Met-tRNA^fMet^, 2 μM IF1, 4 μM IF2, and 2 μM IF3. EM contained 20 μM EF-Tu, 20 μM EF-Ts, 10 μM EF-G, 40 μM tRNA^Leu^, 200 uM Leu, and 1 U/μL Leucyl tRNA synthetase. IM and EM were formed by incubating them at 37 °C for 15 min separately. The RC was formed by mixing IM and EM at 37 °C for 5 min and then quenched by putting in ice. Additional 4 mM Mg(OAc)_2_ was added to stabilize the RC, which was then purified by ultracentrifugation on a 37% sucrose cushion in a swing-out rotor (S55-S; Sorvall) at 258,000*g* for 2 h at 4 °C. The pellet containing RC was later dissolved in HEPES-polymix buffer (pH 7.5).

### Stopped-flow-based fluorescent peptide release assay

The RCs with Met-Leu-Leu-XXX mRNA, where XXX refers to either stop codons (UAA, UGA, UAG) or a Trp codon (UGG), were prepared as described above. Equal volumes of RC and RF1/RF2 were mixed rapidly in a BioLogic (SFM-4000) micro stopped-flow instrument and the time course of BOP-Met-Leu-Leu tripeptide release was measured by following the change of BOP fluorescence (through a long-pass filter of 590 nm) as a function of time. No fluorescence change was observed when RF1/RF2 was not added confirming that the fluorescence change solely indicated release of the BOP-labeled tripeptide with the RFs (Fig. S3). All release experiments were conducted at least in triplicates and standard error of mean (SEM) was estimated.

### Estimation of the kinetic and accuracy parameters

The rate of peptide release was estimated by fitting the fluorescence curves with double exponential decay function (y = A_1_∗exp(-x/t_1_) + A_2_∗exp(-x/t_2_) + y0) where y is the relative fluorescence; x is the time, A is the amplitude of the individual phases; t is the time constant; y0 is the offset, and 1/t is the rate constant (k). The fast phase accounts 80 to 90% of the total peptide release, whereas 10 to 20% of the peptide release happens in the slow phase, possibly due to unknown heterogeneity in the RC. The apparent rates of the fast phase for varying concentrations of RFs were plotted against RF concentration and fitted to the hyperbolic Michaelis–Menten equation from which the Michaelis–Menten parameters (*k*_*cat*_, *K*_M_, and *k*_*cat*_/*K*_M_) were estimated. The “A value” reflecting accuracy in peptide release by the RFs was derived from the equation A = (*k*_*cat*_/*K*_M_)_cognate_/(*k*_*cat*_/*K*_M_)_near-cognate_. Higher A value indicates higher accuracy. Alternatively, nondiscriminating codons produce A value close to 1.

### Thermal melting of mRF2 and RF2 followed by CD and SYPRO-orange fluorescence

CD was used to monitor the change in the secondary structure of the RF2 variants on thermal denaturation. For that, 5 μM mRF2 or RF2 in Phosphate buffered saline (PBS) (pH 7.3) was added in a JASCO J-1500 CD spectrometer. The mean residue ellipticity at 220 nm (θ) was recorded with increase in temperature ranging between 14 °C and 95 °C ramping 0.5 °C at every 5 s. The fraction of the folded proteins was determined by the following equation:

$${\left(\theta \right)}^{obs\;Temp}-{\left(\theta \right)}^{\text{unfolded}}/{\left(\theta \right)}^{\text{folded}}-{\left(\theta \right)}^{\text{unfolded}}$$

where (θ)^obs Temp^ is the ellipticity at a given temperature, (θ)^unfolded^ is the ellipticity at highest temperature, and (θ)^folded^ is the ellipticity at lowest temperature. The experiments were repeated twice and the melting temperature (T_m_) was determined from the transition midpoint.

SYPRO-orange thermal shift assay is an extrinsic fluorescence-based assay to follow the tertiary structure of the proteins. The dye SYPRO-orange binds to the exposed hydrophobic patches of the proteins. Thus, the change in SYPRO-orange fluorescence with increase in temperature reflects access to the otherwise hidden hydrophobic patches during thermal melting. The assay was conducted in a BIO-RAD CFX connect Real-Time PCR system using 10 μM of the RF2s in PBS varying temperature from 15 to 95 °C ramping 1 °C at every 10 s. The first derivatives of the fluorescence scans at 569 nm were plotted against temperature. The T_m_s was determined from the peak values.

## Data availability

All data generated in this study are presented in this article and/or available on request.

## Supporting information

This article contains supporting information.

## Conflict of interest

The authors declare that they have no conflicts of interest with the contents of this article.
