# Supplementary material for: GGQ methylation enhances both speed and accuracy of stop codon recognition by bacterial class-I release factors
Source: J Biol Chem. 2021 Apr 20;296:100681. doi: 10.1016/j.jbc.2021.100681 (PMC8131318; doi:10.1016/j.jbc.2021.100681)
Supplement: Supplemental Figures S1–S3 [file mmc1.pdf]

SUPPORTING INFORMATION

GGQ methylation enhances both speed and accuracy of stop codon recognition by bacterial class-I release factors

Shreya Pundir, Xueliang Ge, and Suparna Sanyal*

Department of Cell and Molecular Biology, Uppsala University, Box-596, BMC, SE-75124 Uppsala, Sweden

*For correspondence: Suparna Sanyal, suparna.sanyal@icm.uu.se

Running Title: GGQ methylation enhances speed and accuracy of RF1/RF2

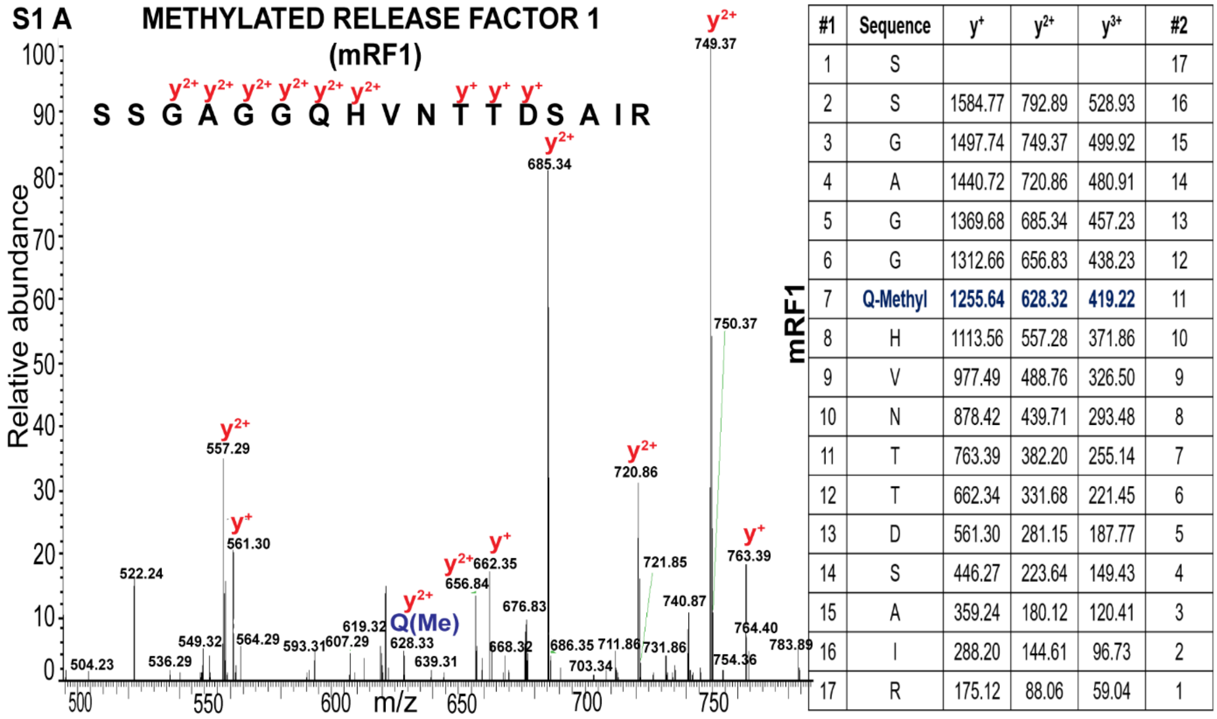

mRF1

#1	Sequence	y+	y2+	y3+	#2
1	S				17
2	S	1584.77	792.89	528.93	16
3	G	1497.74	749.37	499.92	15
4	A	1440.72	720.86	480.91	14
5	G	1369.68	685.34	457.23	13
6	G	1312.66	656.83	438.23	12
7	Q-Methyl	1255.64	628.32	419.22	11
8	H	1113.56	557.28	371.86	10
9	V	977.49	488.76	326.50	9
10	N	878.42	439.71	293.48	8
11	T	763.39	382.20	255.14	7
12	T	662.34	331.68	221.45	6
13	D	561.30	281.15	187.77	5
14	S	446.27	223.64	149.43	4
15	A	359.24	180.12	120.41	3
16	I	288.20	144.61	96.73	2
17	R	175.12	88.06	59.04	1

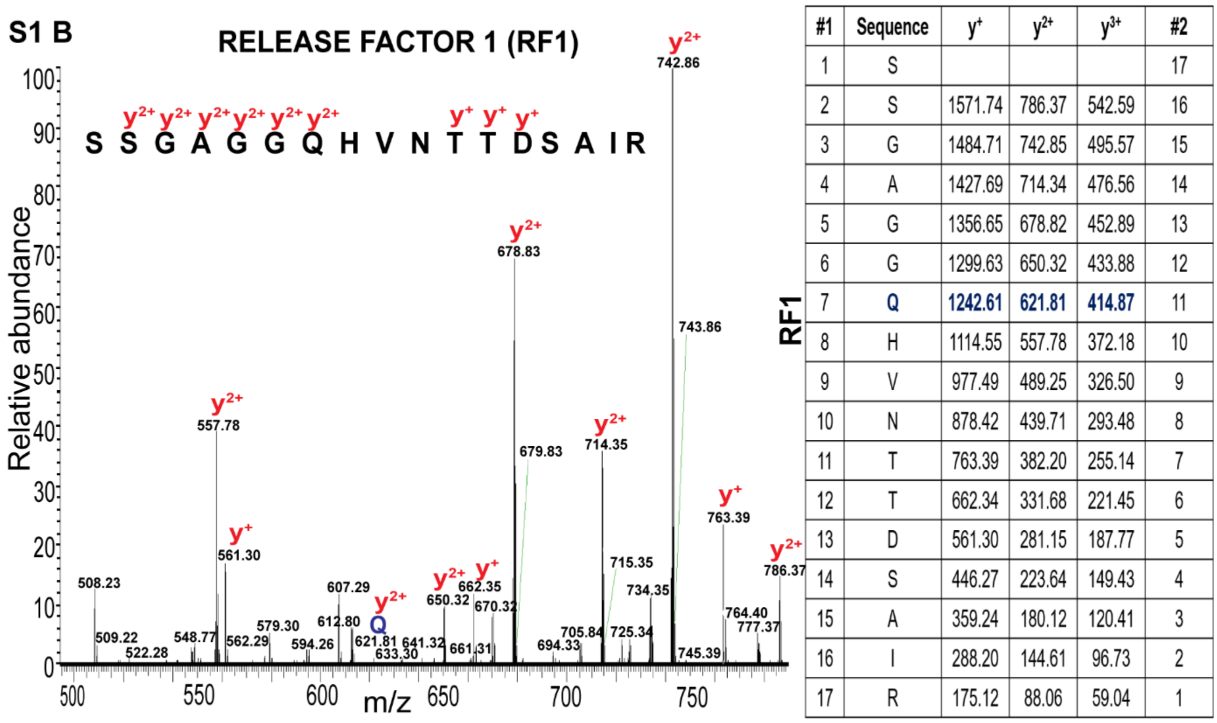

RF1

#1	Sequence	y+	y2+	y3+	#2
1	S				17
2	S	1571.74	786.37	542.59	16
3	G	1484.71	742.85	495.57	15
4	A	1427.69	714.34	476.56	14
5	G	1356.65	678.82	452.89	13
6	G	1299.63	650.32	433.88	12
7	Q	1242.61	621.81	414.87	11
8	H	1114.55	557.78	372.18	10
9	V	977.49	489.25	326.50	9
10	N	878.42	439.71	293.48	8
11	T	763.39	382.20	255.14	7
12	T	662.34	331.68	221.45	6
13	D	561.30	281.15	187.77	5
14	S	446.27	223.64	149.43	4
15	A	359.24	180.12	120.41	3
16	I	288.20	144.61	96.73	2
17	R	175.12	88.06	59.04	1

Supporting Figure S1. Mass spectrometry based confirmation of the methylation status of the RF1 variants. MS/MS spectrum of mRF1 (A) and RF1 (B) obtained after proteolytic digestion of the proteins with Trypsin/Lys-C Mix (Promega). Mass analysis with MaxQuant 1.5.1.2 software as well as Proteome Discoverer 1.4 (Thermo Fisher Scientific) shows 100% methylation on Glutamine 235 residue in mRF1 (m/z: 628.32) (A), while no methylation on Glutamine 235 is seen in RF1 (m/z: 621.81) (B).

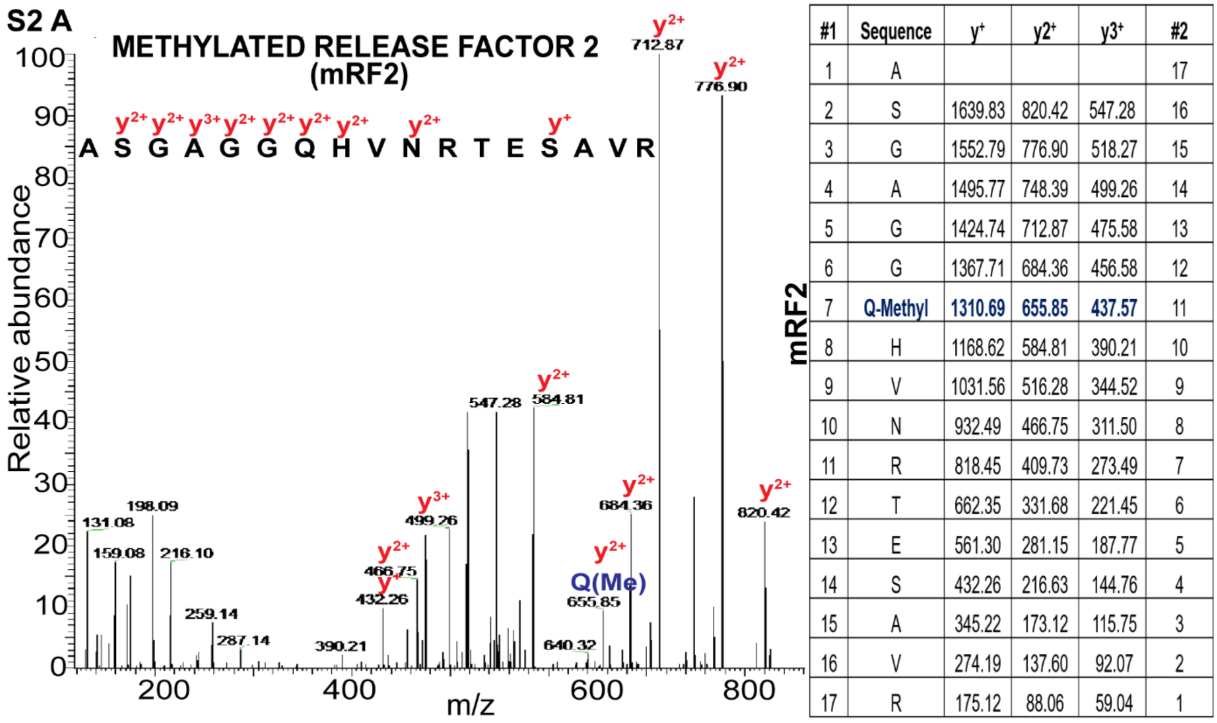

mRF2

#1	Sequence	y⁺	y2⁺	y3⁺	#2
1	A				17
2	S	1639.83	820.42	547.28	16
3	G	1552.79	776.90	518.27	15
4	A	1495.77	748.39	499.26	14
5	G	1424.74	712.87	475.58	13
6	G	1367.71	684.36	456.58	12
7	Q-Methyl	1310.69	655.85	437.57	11
8	H	1168.62	584.81	390.21	10
9	V	1031.56	516.28	344.52	9
10	N	932.49	466.75	311.50	8
11	R	818.45	409.73	273.49	7
12	T	662.35	331.68	221.45	6
13	E	561.30	281.15	187.77	5
14	S	432.26	216.63	144.76	4
15	A	345.22	173.12	115.75	3
16	V	274.19	137.60	92.07	2
17	R	175.12	88.06	59.04	1

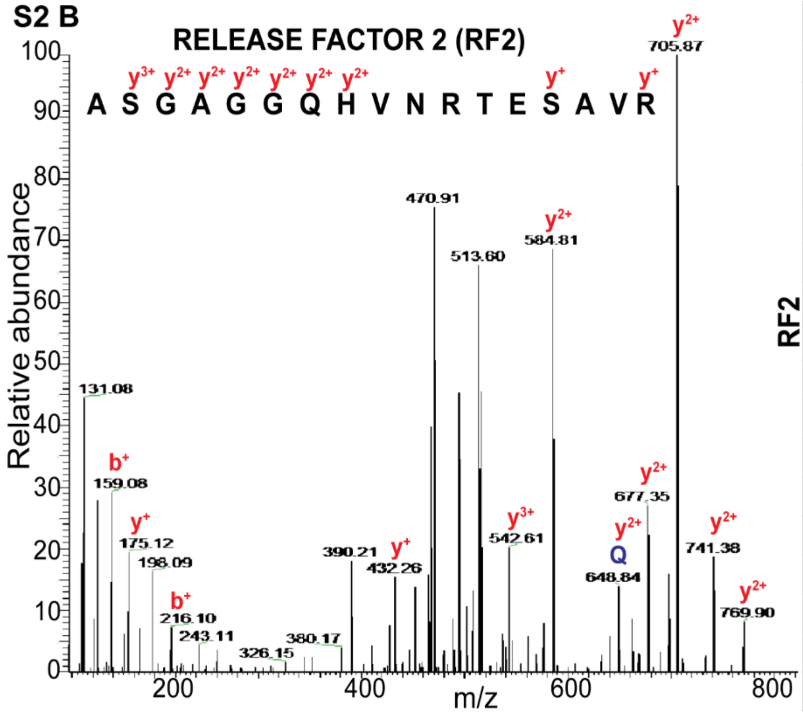

RF2

#1	Sequence	y⁺	y2⁺	y3⁺	#2
1	A				17
2	S	1625.81	813.41	542.61	16
3	G	1538.78	769.90	513.60	15
4	A	1481.76	741.39	494.59	14
5	G	1410.72	705.86	470.91	13
6	G	1353.70	677.35	451.90	12
7	Q	1296.68	648.84	432.90	11
8	H	1168.62	584.81	390.21	10
9	V	1031.56	516.28	344.53	9
10	N	932.49	466.75	311.50	8
11	R	818.45	409.73	273.49	7
12	T	662.35	331.68	221.45	6
13	E	561.30	281.15	187.77	5
14	S	432.26	216.63	144.76	4
15	A	345.22	173.12	115.75	3
16	V	274.19	137.60	92.07	2
17	R	175.12	88.06	59.05	1

Supporting Figure S2. Mass spectrometry based confirmation of the methylation status of the RF2 variants. MS/MS spectrum of mRF2 (A) and RF2 (B). The sample treatment and data analysis were done using the same methods as for the RF1 variants (Supporting Figure S1). The mass analysis shows 100% methylation on Glutamine 252 residue in mRF2 (m/z: 655.85) (A); methylation of Glutamine 252 is totally absent in RF2 (m/z: 648.84) (B).

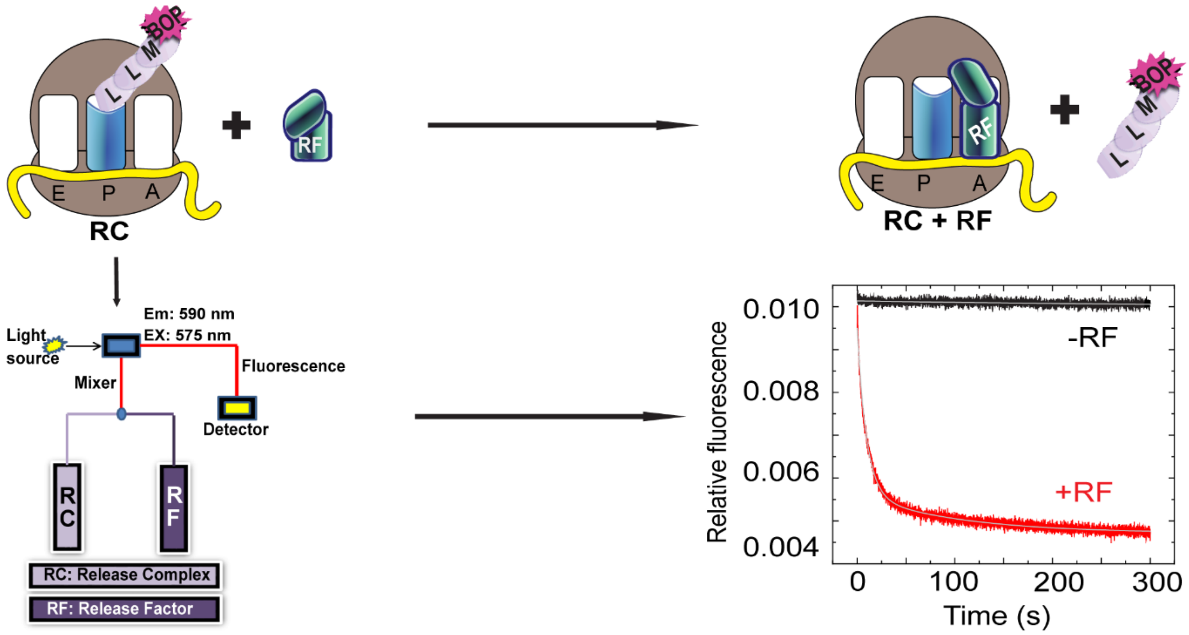

Supporting Figure S3. Stopped-flow based fluorescent peptide release assay. The RC programmed with a Met-Leu-Leu-XXX mRNA (where XXX refers to either UAA, UGA, UAG stop codons or a UGG Trp codon) and with a peptidyl tRNA carrying BOP-Met-Leu-Leu tripeptide in the P-site was prepared as described in Materials and Methods. Equal volumes of RC and RF (variants) were mixed rapidly in a BioLogic (SFM-4000) micro stopped-flow instrument and the time course of BOP-Met-Leu-Leu peptide release was measured by monitoring BOP fluorescence, through a long pass filter of 590 nm. The red curve presents the time course of BOP-Met-Leu-Leu peptide release by 20 μM RF2 on 0.1 μM RC with UAG codon in the A site. The black curve with insignificant fluorescence change presents the time course without any RF, thereby confirming that the change in BOP fluorescence (red curve) certainly indicates peptide release with the RFs.
